# Dual circumflex artery from left main coronary artery: a rare coronary artery anomaly

**DOI:** 10.21542/gcsp.2024.54

**Published:** 2024-12-31

**Authors:** Mohamed M. Elgayar, Asmaa Ahmed, Mohamed Abdelfattah

**Affiliations:** 1 Aswan Heart Centre, Magdi Yacoub Foundation, Aswan, Egypt; 2 Department of Radiology, Al-Azhar University, Cairo, Egypt

## Abstract

Coronary artery anomalies are uncommon in the general population. The most frequently reported anomaly involving the circumflex artery is its origin from the right aortic sinus or the right coronary artery. However dual circumflex artery is an extremely rare anomaly. We report a case of a 79-year-old male patient who was admitted to our centre with a diagnosis of severe aortic stenosis, scheduled for transcatheter aortic valve implantation. Computed tomography angiography revealed dual circumflex arteries: one originating normally from usual bifurcation of the left main coronary artery and an accessory one arising separately from the left main coronary artery stem proximal to the bifurcation.

## Introduction

Coronary artery anomalies are uncommon in the general population, with an estimated incidence of 0.6–1.3% among patients undergoing coronary angiography. These anomalies are often asymptomatic and discovered incidentally^1,2^. One of the most frequently observed coronary anomalies is the left circumflex (LCx) artery arising abnormally from the right sinus of valsalva or or the right coronary artery (RCA). A much rarer variation involves double or dual LCx arteries, originating separately from both the left and right coronary systems^3^. In this report, we present a extremely rare case of a dual LCx arising from left main coronary artery (LMCA).

### Case

A 79-year-old man with a history of chest discomfort was referred to our centre. He had no history of coronary artery disease, or alcohol/drug use. The only risk factor was hypertension. His physical examination revealed an ejection systolic murmur over the upper right sternal area. An ECG showed first-degree heart block and left ventricular (LV) hypertrophy, while tranthoracic echocardiography revealed a hypertrophied LV with normal systolic function and severe calcified aortic stenosis. Computed tomography (CT) angiography was performed in preparation for transcatheter aortic valve implantation (TAVI).

The CT imaging protocol used included two distinct phases. The thoracic phase was retrospectively ECG-gated (30–80% of the RR interval) with five reconstructions (best diastolic, best systolic, and manually at 20%, 30%, and 40% intervals) using 0.6 mm slice thickness, a B26f kernel, and cardiac windowing. The abdominal phase utilized a high-pitch (2.2), non-ECG-gated acquisition with free breathing, reconstructed at one mm slice thickness, a B10f kernel, and CT angiography windowing. Calcium scoring was performed prospectively at 70% of the RR interval with three mm slices using a B35 medium kernel. All scans were conducted on Siemens SOMATOM Definition Flash scanner.

**Figure 1.**
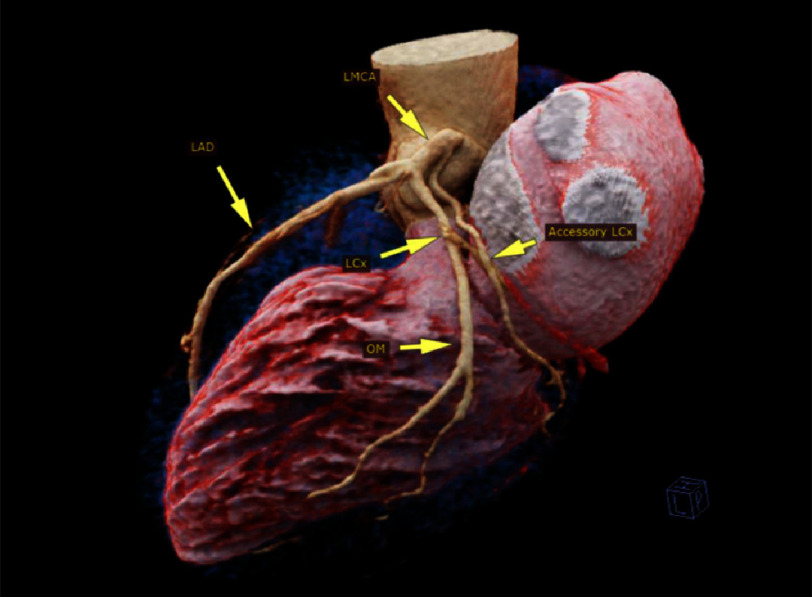
Volume-rendered computed tomography of the heart showing the origin and course of main and acessory circumflex artery. LMCA: left main coronary artery, LAD: left anterior descending, LCx: left circumflex, OM: obtuse marginal.

**Figure 2.**
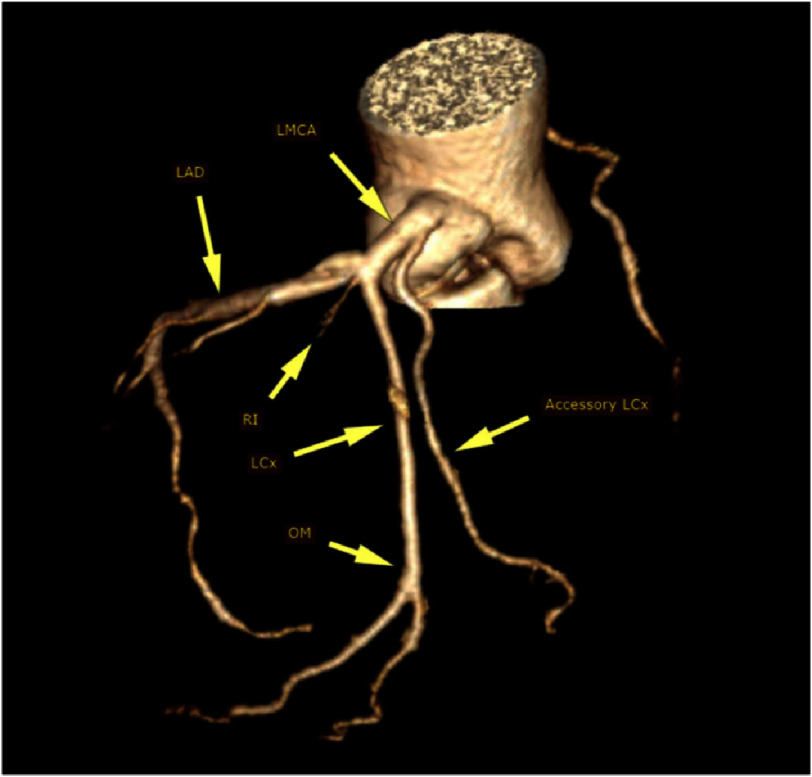
Volume-rendered computed tomography of the coronary tree showing trifurcation of left main coronary artery and acessory circumflex artery. LMCA: left main coronary artery, LAD: left anterior descending, LCx: left circumflex, RI: ramus intermedius, OM: obtuse marginal.

**Figure 3.**
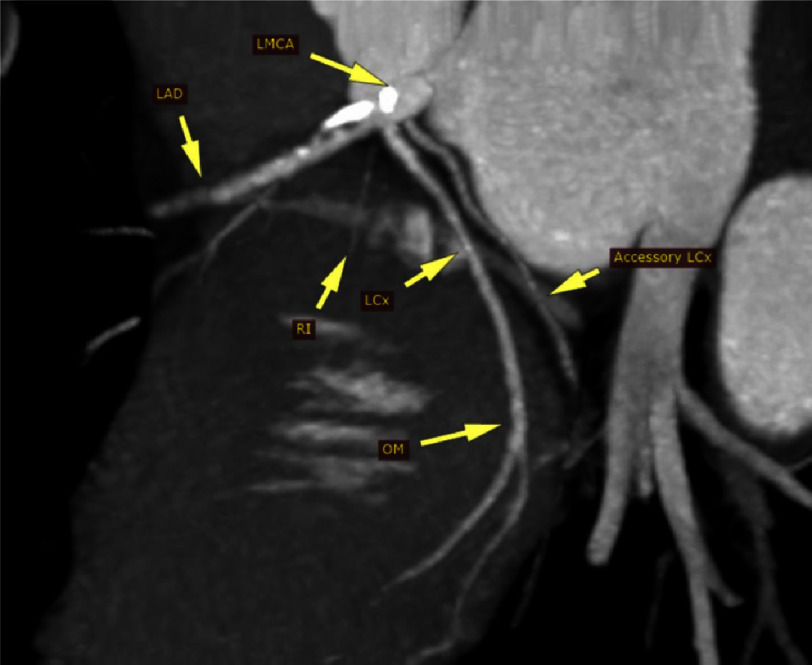
Curved multiplanar reconstructions reformated showing trifurcation of left main coronary artery and acessory circumflex artery. LMCA: left main coronary artery, LAD: left anterior descending, LCx:, RI: ramus intermedius, OM: obtuse marginal.

The imaging showed a thickened trileaflet aortic valve with severe stenosis. The RCA was seen arising from the right coronary sinus. The LMCA arises from the left coronary sinus. It trifurcates into left anterior descending (LAD) artery, a small ramus intermedius (RI) and LCx artery. The LCx runs in the left atriventricular groove (AV) for a short distance then it supplies a bifurcating obtuse marginal (OM) at the lateral LV wall. Another LCx is seen arising from the LMCA stem, 3 mm from its ostium. It runs in the left AV groove, not supplying any side branches and then terminates at the inferior LV wall as a small OM artery (Figures 1, 2 and 3).

## Discussion

The most commonly reported anomaly with respect to LCx artery is its origin from the right aortic sinus or the RCA with a retro-aortic course^4^. While the duplication of the LAD artery has been more frequently described and classified ^5^. Jariwala et al. offered a classification of the LAD into three main groups based on angiographic and autopsy findings^6^. Twin or dual circumflex arteries have been reported originating from both the right and the left coronary artery systems, where the accessory LCx was seen originating from the RCA or the right aortic sinus^3^.

Uğuz et al. presented two cases of dual LCx arteries associated with acute coronary syndrome. In both cases, angiography revealed one LCx originating from the LMCA and the other from the proximal RCA. These cases underscore the clinical significance of dual circumflex arteries, particularly in patients presenting with acute coronary events^7^ Sharma et al. described a rare case of dual circumflex coronary arteries, where the accessory LCx originated directly from the left coronary sinus. This anomaly occurred alongside a normal bifurcation pattern of the left main coronary artery^8^.

In our case acessory LCx originates from left coronary artery proximal to bifurcation into LAD and main Lcx and small RI. To our knowledge, this specific anatomical variation has not been previously documented in the literature.

Certain coronary artery anomalies can lead to chest pain, heart failure, arrhythmia, and even sudden death. These symptoms may arise due to repeated compression of the anomalous artery by a dilated aortic root, the presence of slit-like ostia, or unusual angling caused by the retroaortic course of the LCx artery^9^. Although in our case this anomaly has no clinical significance, it may be important in patients undergoing coronary intervention or cardiac surgery^10^.

## What have we learned?

This case underscores the critical importance of detailed imaging in identifying rare coronary artery anomalies, particularly when planning interventions. The dual origin of the left circumflex artery from the left main coronary artery represents a previously undocumented variant, enriching the spectrum of known coronary anomalies. While this anomaly was asymptomatic in our patient, it holds potential clinical significance, as it may complicate procedures like coronary artery bypass grafting or percutaneous interventions. Accurate pre-procedural imaging, such as multi-detector CT, is therefore essential to identify such variations, avoid intraoperative surprises, and minimize procedural risks.

## Conflicts of Interest

The authors declared no potential conflicts of interest with respect to the research, authorship, and/or publication of this article.

## Funding

The authors received no financial support for the research, authorship, and/or publication of this article.
